# Reduced walking speed at discharge predicts mortality after clinical osteoporotic vertebral fracture: A retrospective cohort study

**DOI:** 10.1007/s11657-026-01686-w

**Published:** 2026-03-16

**Authors:** Hiroyuki Tominaga, Shinobu Uezono, Ichiro Kawamura, Yoshitaka Yamashita, Noboru Taniguchi

**Affiliations:** 1 Department of Orthopaedic Surgery, Graduate School of Medical and Dental Sciences, Kagoshima University, 8-35-1 Sakuragaoka, Kagoshima, 890-8520 Japan; 2 Department of Orthopaedic Surgery, Izumi Regional Medical Center, 4513 Akasegawa, Akune, 899-1611 Japan

**Keywords:** Clinical osteoporotic vertebral fracture (OVF), Osteoporosis, Mortality after vertebral fracture, Walking speed, Sarcopenia, Local kyphosis

## Abstract

***Summary*:**

This study investigated how walking speed affects survival in patients with clinical osteoporotic vertebral fractures. A walking speed below 0.71 m/sec was associated with increased mortality. Walking speed may serve as a simple yet powerful prognostic indicator, highlighting the importance of mobility preservation in fracture management.

**Purpose:**

The number of patients with fractures caused by osteoporosis has increased with the increasing proportion of aging adults in recent years. Osteoporotic vertebral fractures (OVFs) are associated with poor prognosis. Although several reports on the life expectancy of patients with OVFs exist, it is unclear how walking speed affects the life expectancy of patients with clinical OVFs. This study investigated the relationship between walking speed and life expectancy after injury in patients with clinical OVFs.

**Methods:**

A total of 104 patients with new clinical OVFs were conservatively treated with a trunk cast from 2015 to 2017. Lumbar spine and femur bone mineral density (BMD), the Geriatric Nutritional Risk Index (GNRI), walking speed, and spinopelvic parameters were measured. We compared the deceased and survival groups and examined the risk factors influencing survival.

**Results:**

Among the 104 patients included in the study, 67 were women; the median age was 82 years, and the median observation period was 1168 days. Thirty-two patients died during the observation period. The deceased group had a lower GNRI and a slower walking speed at discharge. In addition, imaging revealed more local kyphosis and more calcification of vessels in the deceased group. The risk of death increased when the walking speed after the OVF was less than 0.71 m/sec.

**Conclusion:**

A walking speed of 0.71 m/sec or slower was associated with poor prognosis after clinical vertebral fracture. Nutritional management, kyphotic deformity prevention, and walking speed maintenance are essential for vertebral fracture treatment.

**Supplementary Information:**

The online version contains supplementary material available at 10.1007/s11657-026-01686-w.

## Introduction

The quality of life (QOL) of osteoporosis patients decreases with increasing numbers of vertebral fractures [1], and the sagittal balance of the spine has been reported to be correlated with QOL [2]. Osteoporotic vertebral fractures (OVFs) not only affect patient QOL but also increase the risk of death [3]. An increase in the number of vertebral fractures has been shown to increase the risk of mortality [4]. The 5-year mortality rate for patients with clinically diagnosed vertebral fractures is 28% [5]. Walking speed is a simple and easily obtainable indicator of health in older people [6–9] and tends to improve the predictive ability of the FRAX [10] score. Although walking speed has been shown to predict survival in community-dwelling older adults, patients with clinical OVFs face additional fracture-specific burdens—such as acute back pain, immobilization, kyphotic deformity, and prolonged bed rest—that can further reduce mobility and functional recovery. Consequently, whether discharge-time walking speed can predict survival in OVF patients remains unclear.

Despite the known association between physical activity and walking speed, few studies have investigated the factors affecting prognosis in patients with clinical OVFs in a superaged society. Furthermore, no studies have investigated the relationship between walking speed at discharge and life expectancy following clinical OVFs. Therefore, this study aimed to evaluate postfracture survival by analyzing walking speed, nutritional status, and spinal alignment. We hypothesized that slower walking speed at discharge would independently predict mortality.

## Methods

### Study design

This study was designed as a retrospective cohort study that included consecutive patients with clinical osteoporotic vertebral fractures who were treated conservatively at our institution between 2015 and 2017.

### Participants

This study included 104 patients with new clinical OVFs who were treated conservatively at the hospital in a city where 40% of the population was aged 65 years and older. Patients were released from bed rest as soon as possible after injury and treated with cast immobilization of the trunk, followed by rigid corset treatment. The patients were discharged after rehabilitation, including walking training. We excluded patients who were unable to walk at discharge, patients younger than 65 years, patients who underwent surgery, patients with difficulty assessing pain, patients with deficient bone mass assessment, and patients with other fractures (femur or radius).

### Demographic data

Bone mass, bone metabolism markers (TRACP5b, P1NP, and ucOC), the Geriatric Nutritional Risk Index (GNRI), the revised Hasegawa’s Dementia Scale (HDS-R) [11, 12], grip strength, and walking speed were measured during hospitalization. The 10-m walking speed was measured at a comfortable pace, excluding the acceleration and deceleration phases. Assistive devices were allowed only when required for safe walking, as determined by the physical therapist. Spinopelvic parameters (PI: pelvic incidence, LL: lumbar lordosis) and local kyphosis were measured at discharge by standing continuous thoracolumbar Xp [13, 14]. Spinopelvic parameters are radiographic measurements used to evaluate the sagittal alignment of the spine and pelvis, which are closely related to standing posture and functional balance. These parameters, including pelvic incidence (PI) and lumbar lordosis (LL), provide information on spinal alignment and its compensation mechanisms.

We evaluated thoracoabdominal calcification anterior to the vertebral body via lateral Xp [15–17] and assessed the extent of vascular calcification by the number of vertebral bodies. The survival status of the patients was retrieved from the patients’ medical records or by telephone if the physician had not seen the patient. Using these methods, the survival status of all patients was successfully ascertained within 5 years after fracture; therefore, there was no loss to follow-up for survival outcomes during this period (Supplementary Fig. 1).

### Definitions and treatment of clinical OVFs

We used Xp and magnetic resonance imaging (MRI) to diagnose new vertebral fractures involving low back pain with minor trauma. All patients had vertebral lesions with a low T1 area and a high STIR area on MRI [18]. To evaluate spinal instability, MRI was used to assess the presence of a middle column fracture on the basis of Denis’ three-column spine concept [19]. We analyzed vertebral fractures via a 1.5 T EX-HDX (GE Healthcare). In addition, we classified the novel fracture sites as thoracic (above T9), thoracolumbar (T10-L2) or lumbar (L3-5). Duplicate cases were also included. The number of patients with previous vertebral fractures and new multiple vertebral fractures was also assessed. The number of vertebral fractures was calculated as the total number of vertebral fractures, including new and previous fractures. Bone mineral density (BMD) was assessed via the Discovery DXA system (Hologic, Waltham, MA, USA) and expressed as T-scores on the basis of the reference data from the manufacturer.

Vertebral fractures were conservatively treated by placing the patient in a trunk cast after injury and weaning the patient from the cast as soon as possible. Patients wore a trunk cast for 1 month and then changed to a hard corset, which they wore for a total of 3 months. If osteoporosis medication had not been introduced before the injury, almost all patients were given osteoporosis drugs after admission. Rehabilitation was performed not only in the acute phase but also in the convalescent phase, and the total number of days of hospitalization was calculated.

### Statistics

The Wilcoxon test and Fisher’s exact test were used to compare the two groups, and the Kaplan‒Meier method was used for survival analysis. The risk factors for postfracture mortality were determined via multivariate logistic regression analysis, in which the dependent variable was coded as death = 1 and survival = 0. Odds ratios for age are expressed per 1-year increase, and those for walking speed are expressed per 0.1 m/sec increase. In addition, a multiple regression analysis was performed to identify factors associated with discharge walking speed. *P* values less than 0.05 were considered to indicate statistical significance. Statistical analyses were performed via JMP software (version 16: SAS Institute, Cary, NC, USA).

## Results

### Characteristics of patients with clinical OVFs

The median patient age was 82.5 (77.0–86.0) years, and the median GNRI score was 97.2 (91.2–105.2) points (Table 1). The median T-scores of the lumbar spine and femoral neck were −2.7 and −2.4, respectively. The median length of hospital stay during rehabilitation was 35.5 (23.3–51.0) days. The median walking speed at discharge was 0.71 (0.53–1.04) m/sec (Table 2).

**Table 1:** Comparison of patient characteristics between the deceased group and the surviving group after osteoporotic vertebral fracture

Factor	Total (*N* = 104)	Deceased group (*N* = 32)	Survival group (*N* = 72)	*P* value
Age, y.o	82.5 (77.0–86.0)	84.0 (77.5–86.0)	81.0 (76.3–86.8)	0.32
Male, *n* (%)	37 (35.6%)	15 (46.9%)	22 (30.6%)	0.13
Weight, kg	50.0 (43.3–55.6)	47.8 (42.8–51.1)	51.2 (43.3–58.5)	0.1
Body mass index, kg/m^2^	21.3 (18.9–24.1)	19.2 (18.3–23.4)	21.6 (19.3–24.2)	< 0.05
Geriatric Nutritional Risk Index (GNRI)	97.2 (91.2–105.2)	93.0 (88.5–99.5)	99.9 (93.1–107.4)	< 0.005
Estimated glomerular filtration rate (eGFR) mL/min/1.73 m^2^	61.5 (45.0–78.8)	51.0 (42.0–64.5)	65.0 (51.3–81.0)	< 0.01
The revised Hasegawa’s dementia scale (HDS-R)	23.0 (20.0–27.0)	22.5 (16.0–25.0)	24.0 (20.0–27.3)	0.14
Diabetes, *n* (%)	13 (12.5%)	6 (18.8%)	7 (9.7%)	0.21
Malignant tumor, *n* (%)	12 (11.5%)	4 (12.5%)	8 (11.1%)	0.99
Heart disease, *n* (%)	32 (30.8%)	11 (34.3%)	20 (27.8%)	0.65
Low back pain at admission (NRS)	7 (6–8)	7 (7–8)	7 (6–8)	0.70
Low back pain at discharge (NRS)	2 (2–3)	3 (2–3)	2 (2–3)	0.27

The data are presented as medians (25th quartile‒75th quartile) or n (%)

**Table 2:** Functional, biochemical, and bone-related characteristics in the deceased and surviving groups after osteoporotic vertebral fracture

Factor	Total (*N* = 104)	Deceased group (*N* = 32)	Survival group (*N* = 72)	*P* value
Walking speed at discharge, m/sec	0.71 (0.53–1.04)	0.61 (0.42–0.79)	0.77 (0.57–1.1)	0.01
Walking with independence at discharge, *n* (%)	43 (40.4%)	9 (28.1%)	34 (47.2%)	0.09
Hand grip, kg	16.8 (13.1–27.0)	16.2 (12.4–19.9)	18.9 (14.5–28.3)	0.11
Length of hospital stay, days	35.5 (23.3–51.0)	32.0 (23.3–52.8)	36.0 (22.5–49.5)	0.75
TRACP5b, mU/dL	350.0 (256.0–475.8)	415.0 (268.5–538.0)	333.0 (252.3–433.8)	0.11
P1NP, ng/mL	52.8 (33.8–79.6)	53.1 (42.9–92.8)	48.7 (30.4–73.8)	0.29
ucOC, ng/mL	4.3 (2.9–7.6)	5.3 (3.4–9.1)	3.9 (2.7–7.3)	0.37
Lumbar BMD, g/cm^2^	0.82 (0.69–0.99)	0.85 (0.7–1.0)	0.79 (0.69–0.96)	0.59
Lumbar T-score	−2.7 (−3.5- −1.4)	−2.7 (−3.4- −1.1)	−2.7 (−3.6- −1.5)	0.99
Femoral neck BMD, g/cm^2^	0.64 (0.57–0.73)	0.64 (0.60–0.80)	0.63 (0.56–0.72)	0.35
Femoral neck T-score	−2.4 (−3.1- −1.8)	−2.4 (−2.9- −1.8)	−2.6 (−3.1- −1.8)	0.55
Radius BMD, g/cm^2^	0.55 (0.46–0.69)	0.59 (0.42–0.72)	0.55 (0.46–0.67)	0.98
Radius T-score	−3.4 (−4.5- −2.4)	−3.4 (−4.8- −2.1)	−3.4 (−4.4- −2.5)	0.89

P1NP (N-terminal propeptide of type I procollagen), TRACP 5b (tartrate-resistant acid phosphatase 5b), uc OC (undercarboxylated osteocalcin), The data are presented as medians (25th quartile‒75th quartile) or n (%)

### Comparison between the nonsurviving group and the surviving group

There were 32 deaths during the follow-up period, which lasted a median of 1168 days. The causes of death after injury were pneumonia, including aspiration pneumonia in 7 patients; cardiac disease in 9 patients; malignant tumors in 4 patients; cerebrovascular disease in 3 patients; and unknown disease in 9 patients.

The median ages of the patients in the deceased group and the survival group were 84.0 and 81.0 years (*P* = 0.32), respectively; the median GNRIs were 93.0 and 99.9 (*P* < 0.005), respectively; and the median estimated glomerular filtration rates were 51.0 and 65.0 mL/min/1.73 m^2^ (*P* < 0.01), respectively (Table 1); and the median walking speeds at discharge were 0.61 and 0.77 (*P* = 0.01), respectively (Table 2).

Patients in the deceased group had statistically significant calcification of the aorta over two vertebrae and greater local kyphosis (Table 3).

**Table 3:** Comparison of patient characteristics according to radiological findings between the deceased group and the survival group

Factor		Total	Deceased group	Survival group	*P* value
Level of vertebral fractures	Thoracic, n (%)	5 (4.4%)	0 (0%)	5 (6.9%)	0.32
	Thoracolumbar, n (%)	83 (72.8%)	27 (84.4%)	56 (77.8%)	0.60
	Lumbar, n (%)	26 (22.8%)	7 (21.9%)	19 (26.4%)	0.81
History of previous vertebral fracture, n (%)		33 (31.7%)	7 (21.9%)	26 (36.1%)	0.18
New multiple vertebral fractures, n (%)		17 (16.3%)	6 (18.8%)	11 (15.3%)	0.77
Number of new and old fractures ≥ 2, n (%)		46 (44.2%)	11 (34.4%)	35 (48.6%)	0.20
Number of new and old fractures ≥ 3, n (%)		21 (20.2%)	4 (12.5%)	17 (23.6%)	0.29
Local kyphosis, °		6 (4–8)	6.5 (5.0–13.8)	5 (4–7)	< 0.05
LL, °		35.1 (28.8–42.4)	31.7 (25.1–39.9)	36.6 (30.1–43.7)	0.07
|PI-LL|, °		15.7 (7.8–32.1)	13.3 (1.5–34.8)	19.4 (8.8–31.9)	0.32
Aortic calcification of vertebrae, n		1 (0–2)	2 (0–3)	1 (0–2)	< 0.01
Middle column fracture, n (%)		12 (11.5%)	3 (9.4%)	9 (12.5%)	0.75

The data are presented as medians (25th quartile‒75th quartile) or n (%)

Hand grip strength, bone metabolic markers, osteoporosis treatment, BMD, and spinopelvic parameters did not significantly differ between patients in the deceased group and those in the surviving group (Tables 2–4).

**Table 4:** Comparison of osteoporosis medications used during hospitalization after osteoporotic vertebral fracture between the deceased and surviving groups

Factor	Total	Deceased group	Survival group	*P* value
History of osteoporosis treatment prior to admission, n (%)	22 (21.2%)	5 (15.6%)	17 (23.6%)	0.44
Bisphosphonate, n (%)	56 (53.9%)	18 (56.3%)	38 (52.8%)	0.83
Teriparatide, n (%)	31 (29.8%)	8 (25%)	23 (31.9%)	0.64

The data are presented as n (%)

### Risk factors influencing life expectancy after fracture

The mortality rate after vertebral fracture was 10.4% at one year and 29.8% at five years.

Male sex and reduced walking speed affected the prognosis of patients after OVF and were found to be risk factors influencing life expectancy after fracture (Table 5).

**Table 5:** Risk factors for death after osteoporotic vertebral fracture

Parameter	Univariate		Multivariate	
	OR (95% CI)	*P* value	OR (95% CI)	*P* value
Age	1.02 (0.97–1.07)	0.50	0.97 (0.91–1.03)	0.29
Male sex	2.01 (0.85–4.75)	0.11	2.94 (1.08–8.0)	< 0.05
GNRI < 92	3.35 (1.37–8.35)	< 0.01	2.41 (0.89–6.51)	0.08
Walking speed, m/sec	0.82 (0.70–0.95)	< 0.01	0.77 (0.62–0.92)	< 0.01

Univariate and multivariate logistic regression analyses for mortality, Odds ratios for continuous variables are presented per 1-year increase in age and per 0.1 m/sec increase in walking speed

The cutoff value of walking speed in terms of affecting life expectancy after the OVF was 0.71 m/sec, with a sensitivity of 68.7% and an area under the curve (AUC) of 0.67 (Supplementary Fig. 2).

**Fig. 1:**
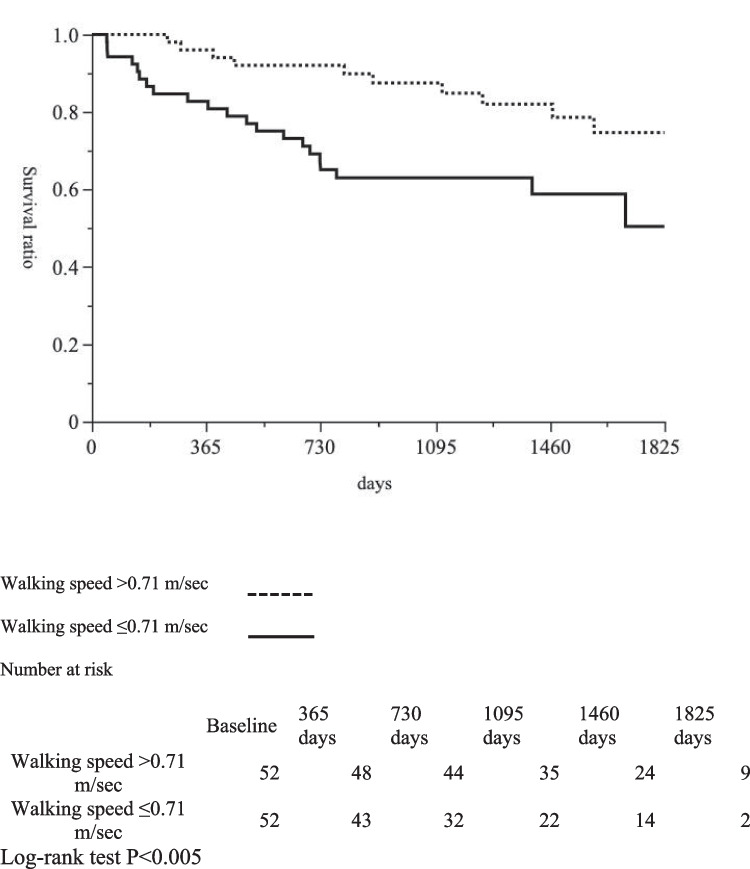
Kaplan–Meier survival curves of the mortality of osteoporotic vertebral fracture patients with a walking speed less than 0.71 m/sec

We defined a cutoff value of 0.71 m/sec or less for walking speed as a risk factor affecting life expectancy after vertebral fracture. Kaplan–Meier survival curves revealed that the mortality rate was greater in patients with a walking speed of 0.71 m/sec or less at discharge (*P* < 0.005) (Fig. 1). In sex-stratified exploratory analyses, slower discharge walking speed was associated with higher postfracture mortality, particularly in men, as shown by Kaplan–Meier analysis (Supplementary Fig. 3). In multivariable analyses, decreased walking speed was identified as an independent predictor of mortality in women, whereas no significant predictors were identified in men, likely reflecting limited statistical power and model instability due to the small number of events in sex-stratified analyses (Supplementary Table 1). Even at three years after vertebral fracture, mortality was significantly greater in the slower walking speed group (Table 6). Finally, to explore the factors associated with walking speed in this cohort, we performed a multiple regression analysis with walking speed as a continuous variable. Older age and a greater local kyphotic angle at the fractured vertebral body were identified as independent factors associated with slower walking speed (Supplementary Table 2).

**Table 6:** Associations between walking speed and mortality three years after vertebral fracture

Factor	Slower walking speed group (Walking speed ≤ 0.71 m/sec)	Faster walking speed group (Walking speed > 0.71 m/sec)	*P* value
Mortality at three years after vertebral fracture, n (%)	16 (30.8%)	6 (11.5%)	0.03

## Discussion

This study revealed that patients with a walking speed of less than 0.71 m/sec after an OVF have a poor life expectancy. This is the first report to provide a cutoff value for walking speed in terms of prognosis after OVF. There are various reports on mortality after OVFs, but few reports on this topic exist. Johnell et al. [5] reported a 1-year mortality rate of 28% and a 5-year mortality rate of 72%. Few 3-year survival analyses have been performed, and the mortality rate in Japan is relatively low compared with that reported previously [20]. A possible explanation is that more than 90% of elderly patients hospitalized with spine fractures receive conservative treatment [21]. Discharge to a rehabilitation facility after vertebral fracture has been reported to reduce postfracture mortality [22], and lower mortality in Japan may reflect early mobilization and comprehensive inpatient rehabilitation, which are common in conservative management. The 1-year and 5-year mortality rates in that study were 10.4% and 29.8%, respectively.

### Risk factors for mortality after OVF

Previous studies reported that older age, male sex, malnutrition, and mobility were associated with increased mortality after OVF [23–25]. Bisphosphonate treatment is associated with decreased mortality in patients after OVF [20]. BP medication is beneficial not only for preventing fractures but also for preventing mortality after OVF. The lack of change in life expectancy associated with osteoporosis medications in this study may be due to the high rate of drug intervention. Twenty-two patients (21%) were treated for osteoporosis before admission, and 78 patients (75%) were treated for osteoporosis after admission. In our study, as in previous studies, malnutrition was a risk factor according to univariate analysis, and male sex was a risk factor according to multivariate analysis. Recently, we identified reduced walking speed at discharge as a risk factor for mortality after OVF.

Additionally, patients who died from OVFs had more vascular calcification. Patients with artery calcification may be at greater risk of vertebral fracture [26]. Aortic calcification is associated with a greater risk of mortality [17].

### Relationship between OVFs and walking speed

Bed rest contributes to decreased walking speed [27]. Multiple vertebral fractures are not significantly associated with low back pain but are significantly associated with reduced walking ability in women [28]. Poor lumbar spine alignment leads to back pain and impaired QOL [29].

In this study, the deceased group had more cases of local kyphosis. Preventing multiple vertebral fractures and avoiding kyphotic deformities are essential to maintaining the ability to walk. Kyphoplasty reduces the kyphotic wedge angle, whereas vertebroplasty restores the height of the vertebral body in OVFs [30].

Since the optimal conservative treatment for individuals with OVFs has not been clearly defined or standardized, it is not possible to conclude that vertebral augmentation techniques are superior to conservative management [31–33].

In this study, we attempted to wean patients from bed rest as early as possible by immobilizing them with a trunk cast.

Patients were hospitalized after clinical vertebral fractures and were discharged or transferred to the hospital after adequate rehabilitation to improve their walking speed. Nevertheless, walking speed is a risk factor influencing life expectancy after rehabilitation and should also be considered. Walking speed in older adults is known to be influenced by age-related decreases in muscle strength and mass, multimorbidity, nutritional status, cognitive impairment, and fear of falling, and is considered a global indicator of frailty and overall health [34–36]. A meta-analysis demonstrated that slower walking speed is associated with increased mortality risk in community-dwelling older adults [34], and sarcopenia-related factors such as lower limb muscle strength and physical performance have been highlighted as major determinants of decreased mobility [35]. Moreover, malnutrition and systemic inflammation contribute to impaired walking performance [36].

In patients with osteoporotic vertebral fractures (OVFs), these general frailty-related factors are compounded by disease-specific mechanisms. Acute vertebral pain, spinal microinstability, sagittal imbalance due to local kyphosis, and enforced immobilization during the acute phase may significantly restrict mobility and accelerate disuse. In our cohort, older age and greater local kyphosis were independently associated with slower discharge walking speed, suggesting the combined impact of frailty and spinal deformity. These findings underscore the importance of early mobilization and prevention of kyphotic progression to maintain walking and improve postfracture outcomes in OVF patients. Given these findings, targeted rehabilitation strategies may help counteract the decline in walking speed and prevent postural deformity after OVFs.

### OVFs and rehabilitation

Dorsal muscle movement therapy can reduce thoracic kyphosis [37].

Postmenopausal osteoporosis combined with low-impact back muscle exercise therapy increases the incidence of lumbar kyphosis by approximately 3% [38]. Vibration stimulation by whole-body vibration machines leads to less bone density loss and improved QOL in postmenopausal women [39].

In situations where osteoporosis is treated or external immobilization is performed, exercise therapy to maintain alignment may increase life expectancy. A walking speed of 0.71 m/sec may be a prognostic indicator for successful rehabilitation after an OVF.

### Limitations

This study has several limitations. First, we did not evaluate the time spent on rehabilitation during hospitalization. However, we started lower limb strength training during bed rest after admission, and patients were not discharged until they could walk. Second, we could not assess whether the patients lived alone after discharge, which may affect the performance of household chores and the ability to return for hospital visits. Third, whether the patient was still taking osteoporosis medications may have played a role in the prognosis, but we were unable to evaluate the effect of continuing osteoporosis medication during the follow-up period. Fourth, sarcopenia may be related to life expectancy, but in this study, we could not measure the skeletal muscle mass of the limbs. However, life expectancy and grip strength were not related, suggesting the importance of walking speed. In addition, renal function and cognitive status (e.g., estimated glomerular filtration rate and Hasegawa Dementia Scale–Revised scores) may represent potential residual confounding factors for walking speed, but these variables were not included in the multivariable analyses. Fifth, this study did not evaluate preinjury frailty. Sixth, the sample size was relatively small (N = 104; 32 deaths). However, effect sizes with 95% confidence intervals and multivariable analyses were provided to strengthen the interpretation. Furthermore, this cohort represented consecutive, single-center patients within a defined period, reflecting real-world clinical practice. Finally, we were not able to evaluate the whole spine with continuous Xp while standing for all patients. Instead, all the samples were taken from the thoracolumbar spine to the hip while the patient was in the standing position.

## Conclusion

The risk factors for mortality after OVF were male sex and reduced walking speed at discharge. A walking speed of 0.71 m/sec may be a prognostic indicator for successful rehabilitation after an OVF.

## Supplementary Information

Below is the link to the electronic supplementary material.

ESM 1 (DOCX 24.9 KB)

ESM 2 (DOCX 54.7 KB)

ESM 3 (DOCX 71.8 KB)

ESM 4 (DOCX 19.1 KB)

ESM 5 (DOCX 18.4 KB)

## Data Availability

The datasets utilized and/or examined in this study can be obtained from the corresponding author upon reasonable request.

## Abbreviations

*OVF*: Osteoporotic vertebral fracture
*BMD*: Bone mineral density
*GNRI*: Geriatric Nutritional Risk Index
*HDS-R*: Revised Hasegawa’s Dementia Scale
*QOL*: Quality of life
*TRACP5b*: Tartrate-resistant acid phosphatase 5b
*P1NP*: Procollagen I N-terminal peptide
*ucOC*: Undercarboxylated osteocalcin
*PI*: Pelvic incidence
*LL*: Lumbar lordosis
*YAM*: Young adult mean
*AUC*: Area under the curve
*BP*: Bisphosphonate

## References

[1] Silverman SL, Minshall ME, Shen W, Harper KD, Xie S, Health-Related Quality of Life Subgroup of the Multiple Outcomes of Raloxifene Evaluation Study (2001) The relationship of health-related quality of life to prevalent and incident vertebral fractures in postmenopausal women with osteoporosis: results from the multiple outcomes of raloxifene evaluation study. Arthritis Rheum 44:2611-2619 10.1002/1529-0131(200111)44:11<2611::aid-art441>3.0.co;2-n 11710717

[2] Imagama S, Hasegawa Y, Matsuyama Y, Sakai Y, Ito Z, Hamajima N, Ishiguro N (2011) Influence of sagittal balance and physical ability associated with exercise on quality of life in middle-aged and elderly people. Arch Osteoporos 6:13–20 22207875 10.1007/s11657-011-0052-1 PMC3235276

[3] Center JR, Nguyen TV, Schneider D, Sambrook PN, Eisman JA (1999) Mortality after all major types of osteoporotic fracture in men and women: an observational study. Lancet 353:878–882 10093980 10.1016/S0140-6736(98)09075-8

[4] Ensrud KE, Ewing SK, Cawthon PM et al (2009) A comparison of frailty indexes for the prediction of falls, disability, fractures, and mortality in older men. J Am Geriatr Soc 57:492–498 19245414 10.1111/j.1532-5415.2009.02137.x PMC2861353

[5] Johnell O, Kanis JA, Oden A, Sernbo I, Redlund-Johnell I, Petterson C, De Laet C, Jonsson B (2004) Mortality after osteoporotic fractures. Osteoporos Int 15:38–42 14593451 10.1007/s00198-003-1490-4

[6] Studenski S, Perera S, Patel K et al (2011) Gait speed and survival in older adults. JAMA 305:50–58 21205966 10.1001/jama.2010.1923 PMC3080184

[7] Master H, Neogi T, Callahan LF et al (2020) The association between walking speed from short- and standard-distance tests with the risk of all-cause mortality among adults with radiographic knee osteoarthritis: data from three large United States cohort studies. Osteoarthritis Cartilage 28:1551–1558 32861851 10.1016/j.joca.2020.08.009 PMC7722103

[8] Liu Y, Xu L, Xu Y, Chen T, Zhu G, Chen Y (2024) Dose-response association between walking speed and all-cause mortality: a systematic review and meta-analysis of cohort studies. J Sports Sci 42:1313–1322 39133765 10.1080/02640414.2024.2390302

[9] Zhang F, Wang H, Bai Y, Huang L, Zhong Y, Li Y (2025) Gait speed and all-cause mortality in whole-spectrum chronic kidney disease: a systematic review and meta-analysis included 6217 participants. J Cachexia Sarcopenia Muscle 16:e13739 39991779 10.1002/jcsm.13739 PMC11848591

[10] Lundin H, Saaf M, Strender LE, Nyren S, Johansson SE, Salminen H (2017) Gait speed and one-leg standing time each add to the predictive ability of FRAX. Osteoporos Int 28:179–187 27844133 10.1007/s00198-016-3818-x PMC5206249

[11] Imai Y, Hasegawa K (1994) The revised Hasegawa’s dementia scale (HDS-R)-evaluation of its usefulness as a screening test for dementia. East Asian Arch Psychiatry 4:20

[12] Jeong JW, Kim KW, Lee DY et al (2007) A normative study of the revised Hasegawa dementia scale: comparison of demographic influences between the revised Hasegawa dementia scale and the mini-mental Status examination. Dement Geriatr Cogn Disord 24:288–293 17717415 10.1159/000107592

[13] Matsumoto T, Hoshino M, Tsujio T et al (2012) Prognostic factors for reduction of activities of daily living following osteoporotic vertebral fractures. Spine (Phila Pa 1976) 37:1115–1121 22158062 10.1097/BRS.0b013e3182432823

[14] Li Y, Sun J, Wang G (2021) Lumbar lordosis morphology correlates to pelvic incidence and erector spinae muscularity. Sci Rep 11:802 33437009 10.1038/s41598-020-80852-7 PMC7804424

[15] Kauppila LI, Polak JF, Cupples LA, Hannan MT, Kiel DP, Wilson PW (1997) New indices to classify location, severity and progression of calcific lesions in the abdominal aorta: a 25-year follow-up study. Atherosclerosis 132:245–250 9242971 10.1016/s0021-9150(97)00106-8

[16] Okuno S, Ishimura E, Kitatani K et al (2007) Presence of abdominal aortic calcification is significantly associated with all-cause and cardiovascular mortality in maintenance hemodialysis patients. Am J Kidney Dis 49:417–425 17336703 10.1053/j.ajkd.2006.12.017

[17] Wang L, Cheng H, Zou X et al (2021) Prevalence and correlates of cardiovascular calcification and its prognostic effects among patients with chronic kidney disease: results from the C-STRIDE study. Front Public Health 9:762370 35071158 10.3389/fpubh.2021.762370 PMC8771912

[18] Kanchiku T, Taguchi T, Kawai S (2003) Magnetic resonance imaging diagnosis and new classification of the osteoporotic vertebral fracture. J Orthop Sci 8:463–466 12898295 10.1007/s00776-003-0665-3

[19] Denis F (1984) Spinal instability as defined by the three-column spine concept in acute spinal trauma. Clin Orthop Relat Res 189:65–76 6478705

[20] Iida H, Sakai Y, Seki T, Watanabe T, Wakao N, Matsui H, Imagama S (2022) Bisphosphonate treatment is associated with decreased mortality rates in patients after osteoporotic vertebral fracture. Osteoporos Int 33:1147–1154 35022813 10.1007/s00198-021-06264-z

[21] Harada A, Matsuyama Y, Nakano T, Deguchi M, Kuratsu S, Sueyoshi Y, Yonezawa Y, Wakao N, Machida M, Ito M (2010) Nationwide survey of current medical practices for hospitalized elderly with spine fractures in Japan. J Orthop Sci 15:79–85 20151255 10.1007/s00776-009-1411-2

[22] Fields DP, Varga G, Alattar A, Shanahan R, Das A, Hamilton DK, Okonkwo DO, Kanter AS, Forsythe RM, Weiner DK (2024) Preinjury frailty predicts 1-year mortality in older adults with traumatic spine fractures. Neurosurgery 95:676–681 38551355 10.1227/neu.0000000000002913 PMC12245289

[23] McCullough BJ, Comstock BA, Deyo RA, Kreuter W, Jarvik JG (2013) Major medical outcomes with spinal augmentation vs conservative therapy. JAMA Intern Med 173:1514–1521 23836009 10.1001/jamainternmed.2013.8725 PMC4023124

[24] Bouza C, Lopez T, Palma M, Amate JM (2007) Hospitalised osteoporotic vertebral fractures in Spain: analysis of the national hospital discharge registry. Osteoporos Int 18:649–657 17221295 10.1007/s00198-006-0292-x

[25] Maravic M, Taupin P, Roux C (2013) Hospital burden of vertebral fractures in France: influence of vertebroplasty. Osteoporos Int 24:2001–2006 23340949 10.1007/s00198-012-2264-7

[26] Fusaro M, Tripepi G, Plebani M et al (2021) The vessels-bone axis: Iliac artery calcifications, vertebral fractures and vitamin K from VIKI study. Nutrients 13:3567 34684568 10.3390/nu13103567 PMC8539275

[27] Coker RH, Hays NP, Williams RH, Wolfe RR, Evans WJ (2015) Bed rest promotes reductions in walking speed, functional parameters, and aerobic fitness in older, healthy adults. J Gerontol A Biol Sci Med Sci 70:91–96 25122628 10.1093/gerona/glu123 PMC4342684

[28] Yoshimura N, Muraki S, Oka H, Tanaka S, Kawaguchi H, Nakamura K, Akune T (2015) Factors affecting changes in the serum levels of 25-hydroxyvitamin D: a 3-year follow-up of the ROAD study. Osteoporos Int 26:2597–2605 26089134 10.1007/s00198-015-3184-0

[29] Hasegawa K, Okamoto M, Hatsushikano S, Shimoda H, Ono M, Watanabe K (2016) Normative values of spino-pelvic sagittal alignment, balance, age, and health-related quality of life in a cohort of healthy adult subjects. Eur Spine J 25:3675–3686 27432430 10.1007/s00586-016-4702-2

[30] Wang B, Zhao CP, Song LX, Zhu L (2018) Balloon kyphoplasty versus percutaneous vertebroplasty for osteoporotic vertebral compression fracture: a meta-analysis and systematic review. J Orthop Surg Res 13:264 30348192 10.1186/s13018-018-0952-5 PMC6196425

[31] Longo UG, Loppini M, Denaro L, Brandi ML, Maffulli N, Denaro V (2010) The effectiveness and safety of vertebroplasty for osteoporotic vertebral compression fractures. A double blind, prospective, randomized, controlled study. Clin Cases Miner Bone Metab 7:109–113 22460014 PMC3004456

[32] Longo UG, Loppini M, Denaro L, Maffulli N, Denaro V (2012) Osteoporotic vertebral fractures: current concepts of conservative care. Br Med Bull 102:171–189 22130906 10.1093/bmb/ldr048

[33] Longo UG, Loppini M, Denaro L, Maffulli N, Denaro V (2012) Conservative management of patients with an osteoporotic vertebral fracture: a review of the literature. J Bone Joint Surg Br 94:152–157 22323677 10.1302/0301-620X.94B2.26894

[34] Colon-Emeric CS, McDermott CL, Lee DS, Berry SD (2024) Risk assessment and prevention of falls in older community-dwelling adults: a review. JAMA 331:1397–1406 38536167 10.1001/jama.2024.1416 PMC12224174

[35] Cruz-Jentoft AJ, Bahat G, Bauer J et al (2019) Sarcopenia: revised European consensus on definition and diagnosis. Age Ageing 48:16–31 30312372 10.1093/ageing/afy169 PMC6322506

[36] Lorenzo-Lopez L, Maseda A, de Labra C, Regueiro-Folgueira L, Rodriguez-Villamil JL, Millan-Calenti JC (2017) Nutritional determinants of frailty in older adults: a systematic review. BMC Geriatr 17:108 28506216 10.1186/s12877-017-0496-2 PMC5433026

[37] Sinaki M, Itoi E, Wahner HW, Wollan P, Gelzcer R, Mullan BP, Collins DA, Hodgson SF (2002) Stronger back muscles reduce the incidence of vertebral fractures: a prospective 10 year follow-up of postmenopausal women. Bone 30:836–841 12052450 10.1016/s8756-3282(02)00739-1

[38] Hongo M, Itoi E, Sinaki M, Miyakoshi N, Shimada Y, Maekawa S, Okada K, Mizutani Y (2007) Effect of low-intensity back exercise on quality of life and back extensor strength in patients with osteoporosis: a randomized controlled trial. Osteoporos Int 18:1389–1395 17572835 10.1007/s00198-007-0398-9

[39] Sen EI, Esmaeilzadeh S, Eskiyurt N (2020) Effects of whole-body vibration and high impact exercises on the bone metabolism and functional mobility in postmenopausal women. J Bone Miner Metab 38:392–404 31897748 10.1007/s00774-019-01072-2

