# Supplementary material for: Reduced walking speed at discharge predicts mortality after clinical osteoporotic vertebral fracture: A retrospective cohort study
Source: Arch Osteoporos. 2026 Mar 16;21(1):51. doi: 10.1007/s11657-026-01686-w (PMC12992475; doi:10.1007/s11657-026-01686-w)
Supplement: Supplementary file 1 — (DOCX 24.9 KB) [file 11657_2026_1686_MOESM1_ESM.docx]

**Supplementary Figure 1.** Flowchart of patient inclusion and follow-up

Patients hospitalized with clinical osteoporotic vertebral fractures (n = 104)

Patients included in the survival analysis. (n = 104)

Survival status was assessed through medical record review or telephone contact after discharge

Survival status was successfully ascertained within 5 years after fracture for all patients (n = 104)

No loss to follow-up (follow-up rate: 100%)
