# Supplementary material for: Reduced walking speed at discharge predicts mortality after clinical osteoporotic vertebral fracture: A retrospective cohort study
Source: Arch Osteoporos. 2026 Mar 16;21(1):51. doi: 10.1007/s11657-026-01686-w (PMC12992475; doi:10.1007/s11657-026-01686-w)
Supplement: Supplementary file 2 — (DOCX 54.7 KB) [file 11657_2026_1686_MOESM2_ESM.docx]

**Supplementary Figure 2.** Receiver operating characteristic (ROC) curve for determining the cutoff value of walking speed

Sensitivity

1-Specificity

Cut off: 0.71 m/sec

AUC: 0.67

Sensitivity: 68.7%
