# Supplementary material for: Reduced walking speed at discharge predicts mortality after clinical osteoporotic vertebral fracture: A retrospective cohort study
Source: Arch Osteoporos. 2026 Mar 16;21(1):51. doi: 10.1007/s11657-026-01686-w (PMC12992475; doi:10.1007/s11657-026-01686-w)
Supplement: Supplementary file 3 — (DOCX 71.8 KB) [file 11657_2026_1686_MOESM3_ESM.docx]

**Supplementary Figure 3.** Kaplan–Meier survival curves showing sex-specific mortality in patients with osteoporotic vertebral fractures, comparing those with walking speeds below and above 0.71 m/sec

1. Female

Log-rank test P=0.08

1. Male

Log-rank test P<0.05

Walking speed >0.71 m/sec

Walking speed ≤0.71 m/sec
