# Supplementary material for: Reduced walking speed at discharge predicts mortality after clinical osteoporotic vertebral fracture: A retrospective cohort study
Source: Arch Osteoporos. 2026 Mar 16;21(1):51. doi: 10.1007/s11657-026-01686-w (PMC12992475; doi:10.1007/s11657-026-01686-w)
Supplement: Supplementary file 4 — (DOCX 19.1 KB) [file 11657_2026_1686_MOESM4_ESM.docx]

**Supplementary Table 1.** Comparison of risk factors for death between females and males after osteoporotic vertebral fracture

| Female (N=17) | **Univariate** | | **Multivariate** | |
| --- | --- | --- | --- | --- |
| **Parameter** | **OR (95% CI)** | ***P* value** | **OR (95% CI)** | ***P* value** |
| Age | 1.01 (0.94-1.09) | 0.84 | 0.96 (0.88–1.05) | 0.39 |
| GNRI <92 | 2.80 (0.84-9.30) | 0.09 | 2.34 (0.65–8.29) | 0.19 |
| Walking speed, m/sec | 0.78 (0.60-0.97) | <0.05 | 0.76 (0.57-0.97) | <0.05 |
| Male (N=15) | **Univariate** | | **Multivariate** | |
| **Parameter** | **OR (95% CI)** | ***P* value** | **OR (95% CI)** | ***P* value** |
| Age | 1.04 (0.97–1.11) | 0.32 | 0.97 (0.88-1.06) | 0.54 |
| GNRI <92 | 3.89 (0.97–17.3) | 0.06 | 2.48 (0.46-14.16) | 0.29 |
| Walking speed, m/sec | 0.77 (0.60-0.95) | <0.05 | 0.77 (0.55-1.01) | 0.06 |

Univariate and multivariate logistic regression analyses for mortality

Odds ratios for continuous variables are presented per 1-year increase in age and per 0.1 m/sec increase in walking speed.

During the study period, 17 deaths occurred among female patients and 15 among male patients. Because of the limited number of events in each sex-stratified analysis, the multivariable logistic regression models may have limited statistical power, and the results should be interpreted with caution.
