# Supplementary material for: Reduced walking speed at discharge predicts mortality after clinical osteoporotic vertebral fracture: A retrospective cohort study
Source: Arch Osteoporos. 2026 Mar 16;21(1):51. doi: 10.1007/s11657-026-01686-w (PMC12992475; doi:10.1007/s11657-026-01686-w)
Supplement: Supplementary file 5 — (DOCX 18.4 KB) [file 11657_2026_1686_MOESM5_ESM.docx]

**Supplementary Table 2. Multiple regression analysis identifying factors associated with walking speed**

| Valuable | β | P value |
| --- | --- | --- |
| Age, y.o. | -0.378 | <0.0001 |
| GNRI | 0.155 | 0.085 |
| Local kyphosis, ° | -0.239 | <0.005 |
| Lumbar lordosis, ° | 0.148 | 0.09 |
| Aortic calcification of vertebrae, n | -0.109 | 0.19 |

β indicates the standardized partial regression coefficient.

Multiple regression analysis was performed to identify factors independently associated with walking speed. Walking speed was used as the dependent variable, and age, the geriatric nutritional risk index (GNRI), the local kyphotic angle, the degree of lumbar lordosis, and the number of cases of vertebral vascular calcification were included as explanatory variables. The standardized regression coefficients (β) and p values were calculated to evaluate the strength of the associations. Statistical analyses were performed using JMP software (SAS Institute Inc., Cary, NC, USA).
